# The legal relevance of a minor patient’s wish to die: a temporality-related exploration of end-of-life decisions in pediatric care

**DOI:** 10.1007/s40656-022-00554-3

**Published:** 2023-01-17

**Authors:** Jozef H. H. M. Dorscheidt

**Affiliations:** 1 Groningen Centre for Health Law, Department of Transboundary Legal Studies, Faculty of Law, University of Groningen, PO Box 716, 9700 AS Groningen, The Netherlands; 2 Department of Public Law, Faculty of Law, Maastricht University, PO Box 616, 6200 MD Maastricht, The Netherlands

**Keywords:** Euthanasia, End-of-life decisions, Pediatrics, Minor patients, Temporality, Health law

## Abstract

Decisions regarding the end-of-life of minor patients are amongst the most difficult areas of decision-making in pediatric health care. In this field of medicine, such decisions inevitably occur early in human life, which makes one aware of the fact that any life—young or old—cannot escape its temporal nature. Belgium and the Netherlands have adopted domestic regulations, which conditionally permit euthanasia and physician-assisted suicide in minors who experience hopeless and unbearable suffering. One of these conditions states that the minor involved must be legally competent and able to express an authentic and lasting wish to die. This contribution is different from other legal texts on end-of-life decisions in modern health care. Foremost, it deals with the role time-bound components play in our views on the permissibility of such decisions with regard to minor patients. While other disciplines provide profound reflections on this issue, from a legal point of view this side has hardly been explored, let alone examined with regard to its relevance for the legal permissibility of end-of-life decisions in pediatrics. Therefore, the manuscript inquires whether there are legal lessons to be learned if we look more closely to temporality-related aspects of these end-of-life decisions, particularly in connection to a minor patient’s assumable ability to choose death over an agonizing existence.

## Introduction

The advances in modern medicine have contributed to a significant increase of curative possibilities to overcome various health disorders. Many of these curative remedies have helped to save the lives of sick children who otherwise would have had little chance of survival. Still, not all children who suffer from serious illnesses are destined to benefit from the progress in medical science. With regard to numerous pediatric disorders no effective treatment options are available, whereas in other cases accessible therapies do not always offer what was expected or hoped for. Consequently, pediatricians sometimes face the dilemma whether or not to continue available care or even to forgo medical treatment, while being aware of the fact that the chances of the sick minor’s recovery are limited either way. At times, pediatricians are even confronted with the inescapable certainty that the life of an incurably ill minor cannot be saved (Dorscheidt, 2013, 2019).

These confrontations show that far-reaching dilemmas in health care are hardly bound to a particular period in human life. At the same time, they illustrate that any life—young or old—cannot escape its temporal nature. In a way, this awareness may cause wonder about how the notion of temporality has special significance in our life. In fact, it even can lead to considerations about whether time-bound components are associated with our views on the permissibility of end-of-life decisions by minor patients. Usually, profound reflections on this issue may be expected from social, psychological, ethical/philosophical or even religious scholars. Yet, from a legal perspective, significant sides to this are not often explored, let alone researched with special reference to the justification of such decisions in pediatrics. This raises the question: are there legal lessons to be learned if we look more closely at the temporality related aspects of these end-of-life decisions, for instance in connection to whether a minor patient’s request to die deserves recognition under the law?

Due to this angle of incidence, this paper is different from those dealing with the ‘solid’ legal problems concerning end-of-life decisions in pediatrics. The main purpose of this manuscript is to explore from a legal point of view several temporality-related aspects of these decisions and to contemplate their significance for the legal assessment of such decisions as well as for the legal review of their medical implementation. Therefore, and for good measure, the article cannot present an overview of current philosophical discourses on issues of temporality, nor will it build upon further developments in these discourses. It can address only some legally relevant thoughts on time-connected facts and circumstances, especially in relation to the decisions mentioned. This is even complicated by the fact that various foundations for the legal thoughts presented are inevitably relative, due to an apparent lack of precedent legal research on the notion of temporality. This may explain the essentially explorative approach adopted.

Nevertheless, to take this curiosity-driven approach is challenging, because the legal perspective tends to use time-related elements only in a specific judicial context, and when the content of the notion concerned is clear, properly demarcated and suited for legal application. As we know, many temporal components of human existence, such as emotional maturity, hardly meet these conditions. At the same time, the legal significance of temporality associated aspects of a normatively sensitive topic, such as a minor person’s capacity to make an accountable end-of-life decision, is likely to remain hidden due to a lack of considerate understanding of their deeper meaning. In fact, one might argue, that some of those aspects deserve more attention than they do in the current legal domain. Hence, this article seeks to cautiously point at some temporality-associated building blocks believed to be determining factors for the legal accountability for end-of-life decisions in pediatrics. Before paying further attention to these building blocks, the text offers preliminary considerations and concise descriptions of current regulations and practices on the thanatic issue mentioned in two European countries: the Netherlands and Belgium. After that, the focus is on whether an incurably sick and severely suffering minor patient is able to choose death in a legally relevant sense, and whether temporality-based aspects of this ability should play a role in the assessment of such a minor’s terminal wish.

## Terminology

In order to appreciate some of the temporality aspects of the legal discourse about end-of-life issues in pediatrics, it is necessary to address a few matters of terminology.

The wording ‘end-of-life decisions in medicine’ is a collective noun for decisions in medical care that lead to the death of a patient (Griffiths et al., 2008; Groenhuijsen, 2006). In some countries, the word ‘euthanasia’ covers a whole range of end-of-life matters, while in others it is reserved for a particular constellation and consequently used in a narrow sense. For purposes of proper legal review, however, a strict definition is necessary. In many (Western) countries, ‘euthanasia’ is defined as the deliberate ending of a person’s life at his or her explicit request, by a third person—usually a physician. This intentional life-ending act involves the administering of a (cocktail of) lethal drug(s), through an injection or infusion, in result of which a competent person, who wished to die, passes away in a course of minutes. Euthanasia differs from physician-assisted suicide (PAS), as the latter involves a less active part of the physician. Here, the physician merely provides appropriate medication, while the patient himself/herself ‘commits’ the life-ending act by ‘consuming’ the thanatic drug(s). Whereas the penalization of euthanasia is connected to the executioner of the life-ending act, the legal inadmissibility of PAS is related to the supply of the medication to be used or to otherwise helping or directing a person to realize his/her wish to die.

To deliberately end the life of a person who did not explicitly request to die does not constitute euthanasia. This specific life-ending act rather amounts to homicide or murder. Besides, such an act is sometimes denominated by a typical wording. Deliberate ending of a severely suffering newborn infant’s life by a physician, for instance, has been referred to as ‘medical neonaticide’ (Dorscheidt, 2005, 2008, 2013).

A legitimate non-treatment decision or abstinent policy is a medical decision to withdraw or to abstain from initiating medical care on admissible professional grounds, such as the inability to realize the treatment’s goal and/or the disproportionality between the ends and means of the treatment. Generally, a patient cannot force a physician to provide medical treatment which is reasonably considered medically futile or obviously unethical.^1^ If the grounds for a non-treatment decision are legally unsound, the physician in charge can be held accountable. In particular circumstances this may lead to disciplinary or even criminal charges against the physician.

Palliative care is referred to by the World Health Organization (WHO) as care that improves the quality of life of patients and their families facing the problems associated with life-threatening illness, through the prevention and relief of suffering by treatment of pain and other problems, physical, psychosocial and spiritual.^2^ In this context, care providers sometimes apply palliative sedation (also known as terminal sedation), which primarily aims at lowering a patient’s conscience in the final phase of his/her life in order to reduce discomfort and awareness of illness-related symptoms. Palliative sedation is part of the regular medical treatment catalogue, which means that it requires the patient’s consent.

## Euthanasia and PAS involving minors: a categorization

In general, the issue of euthanasia or PAS involving minors has rarely been investigated. This may be explained by the broad assumption that these phenomena do not occur that often. Although empirical data on the subject are scarce, there are recorded cases about sick minors whose life was ended with medical assistance. However, the lack of widely accessible official reports on such cases may relate to current domestic legal climates, which usually restrict, if not prohibit, the possibilities to perform euthanasia or PAS in adults, let alone in minors. Consequently, it seems as if there is little cause for maintaining and recording information on thanatic practices involving minors. Additional related factors might be the reluctance among medical professionals to publish information on the incidence and particular circumstances in which lethal actions at the explicit request of a patient—minor or adult—occur, as well as the formal classification attending physicians award to the terminal effects of their medical conducts.

Obviously, the debate on (the permissibility of) euthanasia and PAS at a minor patient’s request may benefit from distinctions made between groups of children. Even though several categorizations are possible, it has been argued that, in view of dealing with the matter of a minor’s decision-making ability, grouping minors according to their communicative abilities would create relevant points of reference. Hence, minors could fall into the following groups:

1. newborn infants (= babies in their first year of life) and very young children without communicative awareness;
2. communicative children younger than 12 years of age, who either express
  - insufficiently discerned views, or
  - sufficiently discerned views.
3. children as of age 12 up to—and including—age 17, who either are
  - incapable to express sufficiently discerned views,
  - able to express a discerned view.

Even though children, as other patient groups in health care, are no homogenous category, a categorization like this can be helpful in further deliberations on the admissibility of euthanasia and PAS at an involved minor patient’s request (Dorscheidt, 2019). Yet, to establish a well-considered position on this sensitive issue is far from easy. It is even more difficult to account for a system under domestic law that regulates this matter, since opinions on this usually differ widely within society (Peleg & Tobin, 2019).

## Dutch and Belgian legislation on euthanasia and PAS involving minors

In but a few jurisdictions, the issue of death on request has been regulated in domestic law. Among the countries to have issued statutory regulations on euthanasia and/or PAS are Canada (Ontario, Quebec), the USA (Oregon, Washington, Montana, Vermont, California, Colorado), Columbia, the Netherlands, Belgium, Luxemburg and Spain. In four of six States in Australia, laws on permitting voluntary assisted dying (VAD) have recently been established,^3^ while Germany’s Constitutional Court has ruled in February 2020^4^ that Article 217 of the German Penal Code, which prohibits the intentional and business-based promotion, granting or supporting of another person’s suicide, must be considered unconstitutional.

The Netherlands and Belgium for their part have consciously adopted legislation that includes a special regime for voluntary death of a minor patient at his/her request. This is remarkable, for to issue regulations in this particular area has not been copied much. At the same time, this qualifies both statutory regimes as useful objects of exploration and reflection on whether temporality-related aspects, for instance, were considered during the drafting process. For that matter, this paragraph explains the main characteristics of both statutory regulations, to identify starting points for subsequent contemplations on the legal significance of temporality-sensitive elements in end-of-life decision-making by a minor patient.

### The Dutch euthanasia act

The Netherlands has been the first country to adopt and consolidate (Otlowski, 1997) an Act of Parliament which defines the exceptional conditions under which acts that end the life of a patient at his/her explicit request can be permissible. After a long and intense societal debate, critically analyzed developments in end-of-life jurisprudence as well as indispensable input by the medical, nursing and pharmaceutic professions, the Dutch legislature adopted the Euthanasia Act (EA), which is valid as of April 2002.^5^

This EA states that ending of life at request of the person involved and assisted suicide remain criminal offences under Articles 293 and 294 of the Dutch Penal Code (PC). However, if a physician performs such an act in accordance with statutory requirements of due care and the performance is afterwards reported to the judicial authorities—more specifically: the local coroner—, the involved physician will probably not to be prosecuted. In that case, this physician, if prosecuted, is believed to successfully invoke a statutory ground for impunity introduced by the EA. Omitting to report a case of euthanasia or PAS that should have been reported constitutes a criminal offence, even if all due care requirements are met.

A reported case is reviewed by one of five Regional Euthanasia Review Committees (RERCs). The Committees are established by law and review the case against the requirements of due care laid down in the EA. These requirements prescribe that the physician in charge must:

- be convinced that the patient’s request is voluntary and well-considered;
- be convinced that the patient’s suffering is unbearable and without prospect of improvement;
- have informed the patient about his/her situation and perspectives;
- in close consultation with the patient be convinced that there is no reasonable alternative to the patient’s situation;
- have consulted at least one other independent physician, who has seen the patient and has stated in writing that the attending physician has satisfied the criteria listed in a. to d. above; and,
- have exercised due medical-pharmaceutical care and attention in the process of ending the patient’s life or assisting in his suicide.

If the RERC concludes that the physician involved has acted in accordance with these requirements, the case is settled. As a result, the Public Prosecutor will not be informed about the case. If the due care requirements have not been met, the local coroner will report the case to the Public Prosecutor’s office. The Board of Procurators-General, the highest body of the Dutch Public Prosecution, ultimately decides whether or not the physician will face a criminal charge.

### The Dutch EA-regime concerning minors

As stated above, the EA contains a special regime concerning end-of-life requests or requests for assisted suicide by minor patients. This regime is based on age categories and holds that such requests may be taken into consideration by a physician if it concerns a competent minor aged between 12 and 16 years. If so, a physician is permitted to comply with that request—of course when all other due requirements are met—provided the parents or otherwise authorized caretakers of this minor can concur with the minor’s wish. Minors below the age of 12 are not covered by the EA. This means that no euthanasia or PAS in this age category can be performed in agreement with the EA-requirements of due care. If the minor patient is aged 16 or 17 years, a physician can comply with the minor’s request as long as the parents or caretakers have been involved in the decision-making process. Consequently, the parents’ or caregivers’ approval to carry out the desired euthanasia or assisted suicide is no legal requirement in this age category. Neither is a permissive court-order or an independent psychological or psychiatric evaluation of the minor’s state of mind. Nevertheless, it is believed to fall within the attending physician’s legal responsibility under the EA to establish the minor’s capacity to weigh his/her involved interests. Besides, in RERC review practice, the requirement of consulting an independent physician not only safeguards the assessment of whether the attending physician has met the statutory requirements, it also involves helping this physician contemplate on the particular death wish before deciding whether or not to grant that request. In this respect, it seems hardly objectionable to consider, even implement prudent additional requirements such as a preceding independent professional assessment of the minor’s mental and emotional capacities. If parents or caretakers would object to such an assessment’s outcome that the minor, all things considered, may be considered to express a matured (enough) request and should therefore be admitted to part from life, they could present the case in court. However, whether this would make things easier for the minor involved is doubtful.

### Available Dutch empirical data

Available case reports disclose that euthanasia and PAS involving minor patients are not frequently practiced in the Netherlands. An inventory over the period of 2002 up to November 2022 shows that 17 cases of euthanasia involving minor patients between the age of 12 and 18 have been reported and reviewed. In 13 of these cases the minors were between 16 and 18 years, while in 2 cases (2017 and 2020), the involved child was between 12 and 16 years old. Two reported cases (2005 and 2022) concerned a child aged 12. In all these cases, the minors’ families were involved in the decision-making process and all understood and respected the minor’s wish to die. In its earlier documentation, the RERC’s reported that prior to 2019 none of the cases prior were considered “inaccurate” (Code of Practice, 2018). In the following years, this careful practice appears to have been maintained. While in 2019, no case concerning a minor was reported (RTE Annual Report, 2021), in 2020 (RTE Annual Report, 2020) and 2021 (RTE Annual Report, 2021) a total of two cases involving a minor patient was reviewed. Both cases were found “accurate” too. Even though the Annual Report of 2022 is not yet available, the RERCs have shared information about one case involving a minor aged 12 and this case was reviewed and considered “accurate” in September 2022.^6^ To illustrate the involved particularities and for reasons of transparency, the RERCs have published several of these cases on their website; available in English and obviously anonymized.^7^ The same goes for the various annual reports referring to the cases concerning minors.

### The Belgian euthanasia act

The Belgian Euthanasia Act (BEA)^8^ entered into force in September 2002 (Nys & Deckers, 2003; De Bondt & Vansweevelt, 2006; Griffiths et al., 2008). The preceding legislative process (Adams, 2002) was, unlike the Dutch one, not substantiated by developments in national jurisprudence. Furthermore, the Belgian Medical Order chose to play no significant role in the legislative process. This is remarkable, given the importance of the medical perspective to the content of the BEA, especially if one considers that most Belgian physicians were not convinced that legislation on this subject should be issued. Moreover, the Belgian Act does not explicitly address PAS, but merely affects euthanasia, even though, ethically and legally, euthanasia and PAS differ only marginally (Nys, 2005). Still, while some observers assumed that the BEA’s formal inapplicability to PAS had political motives, the Federal Control and Evaluation Commission on Euthanasia (FCECE)—established by the Euthanasia Act and charged, among other things, with reviewing reported cases—stated in 2004 that PAS falls within the definition of euthanasia, at least as far as during its execution the statutory terms and conditions are met.

In 2014, an amendment of the BEA Act became valid. A crucial change in the regulation involved the removal of the age restriction, thereby extending the euthanasia regime to minors regardless of their age.^9^ In October 2015, the Belgian Constitutional Court ruled that this extension, codifying the possibility for discerned minors to request for euthanasia, is compatible with the Belgian Constitution and with international human rights law.^10^ However, in October 2022, in its Judgement on Mortier v. Belgium, the European Court of Human Rights (ECtHR) found that Belgium failed to fulfil its procedural obligations under Article 2 (= right to life) of the European Convention on Human Rights, when dealing with a case of euthanasia under the BEA.^11^ In its response to this decision, the FCECE advised the Belgian legislator to adapt the BEA in order to comply with the ECtHR’s signalizations.^12^

The BEA covers two situations of euthanasia. The first relates to a current request, the second to an advance directive. With regard to the first situation, Article 3 paragraph 1 BEA states that a physician who performs euthanasia in agreement with the terms and procedures of the BEA commits no crime, provided the patient who requested to die.

- Has attained the age of majority (= 18 years), or
- is a minor emancipated by a court order, or
- is still a minor, but competent and conscious at the time of his or her request.

Concerning the first two categories, the BEA demands that the involved persons are in a medically hopeless situation of persistent and unbearable physical or mental suffering, which cannot be alleviated and that results from a serious and incurable disorder, caused by illness or accident.

With regard to the competent and conscious (“niet-ontvoogde”) minor, Article 3 paragraph 1 BEA states that the child must be in a medically hopeless situation of persistent and unbearable physical suffering, which cannot be alleviated and leads to death within a foreseeable period of time, as well as results from a serious and incurable disorder, caused by illness or accident (Van Assche et al., 2019). For clarity’s sake: this regime does not cover the possibility that a competent and conscious minor’s wish to die may be granted in case the minor’s situation relates to mental suffering. Furthermore, Article 1 paragraph 7 BEA defines due care requirements for this minor. In case of a request by such a minor, the pediatrician in charge must consult a child- or youth-psychiatrist or a psychologist and explain to this professional the reasons for the consultation. This consultant must look into the minor’s medical file, examine the minor, establish his/her decision-making capacities and attests to all this in writing. The physician must inform the minor’s parents about this consultation and about all other relevant aspects and conditions. Finally, the physician must establish that the parents agree with the minor’s request.

The physician who wishes to perform the requested euthanasia must, previously and in all cases:

- have established that the patient’s desire to die is voluntary, well-considered and expressed repeatedly; the request must be put in writing and be documented in the patient’s medical file;
- be assured that the patient’s physical or mental suffering is persistent;
- have informed the patient about his/her health condition(s) and life expectancy;
- have discussed with the patient: the request, remaining therapeutic options, the possibilities and consequences of palliative care; if necessary, psychological support must have been provided to the patient;
- be convinced that there is no reasonable alternative to the patient’s situation;
- have consulted an independent physician, who has confirmed the patient’s persistent and unbearable suffering, the lack of a reasonable alternative and has reported to that effect;
- have observed at least one month between the written request and the actual thanatic performance; and
- have assured the proper use of appropriate pharmaceutics.

Apart from a special regime regarding euthanasia based on a written advance directive, which, by the way, excludes minors who are not emancipated, the BEA contains a special system of control. Under this system, a physician who has performed euthanasia must report the case to the FCECE. Even though failing to report a case in itself is not penalized under the BEA, it is clear that to meet this obligation is an implicit part of the terms and procedures relevant for impunity. The FCECE reviews the content of a report and decides whether the requirements of careful practice have been met. If the Commission concludes the physician has not complied with these requirements, the case is sent to the Public Prosecutor, who then decides whether the physician in charge will be subjected to criminal proceedings.

As a round up, Article 14 BEA sums up several additional considerations. These reflect that a euthanasia request and an advance directive are not legally binding and that no person can be forced to carry out or to cooperate in performing euthanasia. In addition, Article 15 BEA declares that death resulting from euthanasia, in accordance with the law, counts as a natural death with regard to the execution of (insurance) contracts concluded by the deceased person.

### Available Belgian empirical data

Every two years, the FCECE produces a report on (the amount of) the registration documents reviewed by the Committee and evaluations and clarifications as regards the application of the BEA. The reports also cover suggestions that may lead to alterations of euthanasia policy in Belgium.^13^

Over the period of 2014–2015, no case of euthanasia or PAS concerning competent and conscious minors was reported (FCECE, 2016). However, in the period 2016 up to and including 2021, four cases were reported and reviewed. In September 2016, the first case involving a 17-year old adolescent was confirmed (De Standaard, 2016). A second case was reported that same year (Montero, 2017; Nys, 2017; Van Gool & De Lepeleire, 2017). In 2017, another case occurred (FCECE, 2018). In 2018, no report on minors was received by the FCECE (FCECE, 2019), whereas in 2019 a single reported case was reviewed (FCECE, 2020). According to official press releases, in 2020^14^ and 2021^15^ the FCECE did not review cases of euthanasia or PAS involving a competent and conscious minor. Data with regard to 2022 are not available, yet.^16^ Regrettably, there are no official anonymized records of these four cases to be found at the FCECE’s website or made available elsewhere. Of course, the involved families’ wish for privacy must be respected, but without publication of the main characteristics of these cases and/or the considerations of the FCECE as to why the new due care requirements were met in either case, the public interest of transparency remains insufficiently served.

## Temporality and its legal significance for end-of-life decisions in pediatrics

An intriguing, and from a legal perspective barely studied side to end-of-life decisions in pediatrics relates to whether the issue of temporality holds clues for a careful lawful handling of this topic. Challenging in this regard are questions such as: can awareness of temporality-related considerations be relevant for a proper understanding of the legal rights and wrongs of euthanasia and PAS in pediatric care, and if so, could such considerations play a considerable role in the assessment of the death wish of a minor who severely suffers due to medical conditions? Obviously, authoritative responses to these matters require thorough analyses of related elements of argumentation. At the same time, one should bear in mind that while attempts to voice such responses are made in this paper, it is far from easy to do so convincingly enough. This is not merely because the issue of end-of-life decisions in pediatrics is highly controversial, but also due to the fact that the scientific foundations for viewpoints articulated are less solid than one would expect them to be. The latter is clearly down to an apparent lack of substantial legal research and subsequent strong legal opinions on the elements addressed. In consequence, the views and considerations in this paragraph can only be an impetus for further thoughts and inquiries on the identified temporality-related sides to end-of-life decisions in pediatrics. Yet, however comprehensible these cautions may be, they are not to be regarded as an excuse to avert a serious search for legally important resonances on these temporalities.

### General observations

In contrast to the body of literature on temporality and temporality-related topics (Safranski, 2015; Sherover, 1975), it shows that the significance of temporality for the legal approach of societal issues is but scarcely examined, let alone demonstrated. In a way, this may be explained by the fact that temporal considerations relevant for legal purposes take time predominantly as a linear phenomenon. In consequence, as the legal perspective essentially deals with normative interpretations and evaluations of facts and connections, and whether these are considered right or just, the time-factor requires specification. For, in the end, this factor plays a vital role in the accountability of these interpretations and evaluations.

A current legal issue connected to time is the expiring date of prosecution for certain crimes or liability claims under civil law, meaning that aspired legal actions are limited by a lapse of linear time. Hence, an adequate measurement of time as well as its uniform interpretation is necessary in order to define from which moment on legal effects count. Other legal topics with a temporal connotation are abortion (various countries adopted laws on the termination of pregnancy which include a time limit model in declaring 24 weeks as the limit for legal abortion; Eser & Koch, 2005), the legal maturity of a person, the exercise of certain rights (i.e. the right to vote, to draft a living will/advance directive, to join the army, or the right to autonomously decide about medical treatment, to participate in medical research or to decide on post-mortem donation of one’s organs), the age for legal liability or the minimum age for application of the death penalty. These are set boundaries of calendar age and usually they leave but little room for deviations.

In some jurisdictions, societal issues—such as euthanasia in minors in Belgium or access by children born out of artificial insemination to sperm-donor identifying information in Sweden—are not regulated with reference to a definite age limit, but with reference to an open norm, like ‘the capacity for discernment’ (B) or ‘sufficient maturity’ (SE). This type of norm also relates to a temporal factor, but less explicitly, so that it leaves room for other foundations for interpretation and evaluation. Eventually, such a norm tends to tone down the relevance of the linear understanding of time as a decisive criterion for prudent decision-making.

Essential points of debate regarding the legal acceptance of euthanasia and PAS in minors are the involved minor’s ability to grasp the magnitude of its irrevocable choice as well as the validation of the authenticity of this choice by others. Below, both issues are discussed.

### Euthanasia, PAS and the competent minor

The concept of a child’s ability to reach an accountable decision is widely investigated. Apart from the legal, ethical or philosophical perspective, this ability has been explored within the fields of sociology, developmental psychology and child psychiatry (Hein, 2015; Dorscheidt & Hein, 2018; Breeuwsma & van Geert, 2019). The capacity of minors to decide on issues in medical care deserves special attention, as it does not merely involve the functioning of mental faculties and skills to determine the essence and meaning of complex information, but also the proficiency to develop a lucid and preferably steady personal view on matters, which are far from easy to deal with. To have a view is one thing, to convince yourself and others of its genuineness and righteousness is quite another. When it comes to determining the authenticity and sincerity of a death wish expressed by a minor who seeks existential relief, it is reasonably necessary to proceed farsighted and with appropriate caution.

Generally, the notion of a child’s ability to make a legally sound decision relates to four mental abilities (Grisso et al., 1995; Welie, 2008; Hein et al., 2015). These involve:

- *understanding* the child involved can grasp information relevant for the decision to be made;
- *appreciation* the child involved can acknowledge and value the consequences of the possible options for his or her own situation;
- *deciding* the child involved can process relevant information and arrive at a choice; and,
- *reasoning* the child can logically relate an expressed choice to existing circumstances and to-the-point arguments.

Even though these abilities offer tools for examining the level of a child’s decision-making talent in the context of medical care, to assess a demonstration of these abilities will probably still occur with a reference to chronological age. While it is imaginable that an objectifiable and workable proof of these abilities can be an alternative to chronological age as the decisive legal norm for competent (enough) decision-making, the question is whether such an instrument is likely to be developed or even agreed upon. Therefore, and at least for the time being, there is good cause to suggest that reliably establishing competent decision-making by minors may profit from a combined use of chronological as well as non-chronological standards, rather than from lending authority to the chronological one only and leaving the added value of non-chronological standards unutilized. Yet, in order to show the benefits of applying non-chronological standards it is necessary to identify as well as study them in view of their possible legal revenues.

Besides, it must be noted that the legal acceptance of sound decision-making, also by minors, does not depend on the outcome of a decision-making process, nor on whether the most correct decision was found. Fairly decisive is the extent to which a demonstrated decision-making process can be rationally reproduced. Eventually, this means that a legally relevant ability of a child to make a sound decision preferably constitutes a *sufficient* ability, rather than an *ultimate* ability to decide. Moreover, this ability is not to be measured by the extent to which the decision found could be wrong. Irrational and erroneous choices are made every day, but that does not mean that everyone who makes them by definition lacks legally relevant decision-making capacities. Only if a person’s process of choosing is beyond serious comprehension and is hardly in any sense conceivable, there is room for reasonable doubt about his or her decision-making qualities (Dorscheidt, 2018).

Unfortunately, that is from a scientific point of view, it turns out that studies on the *sufficient* ability of minors to decide about ending their life due to hopeless and unbearable suffering instigated by serious and incurable disorders have not been conducted.^17^

### Temporality and evolvement of the minor person

Assuming that a minor’s legal competency can be interpreted as a *sufficient* ability to reach a reasonable and accountable decision, the question now is whether temporal factors affect this ability, and if so, to what extent an awareness of these factors can determine our judgement of a refined display of this ability. For this purpose, it is required to define which areas of this ability are subjected to temporal influences.

Obviously, the level of understanding of received information, of consequences of available options and of argumentations to support or to decline choices made will evolve in the course of maturation, so that this decisional ability develops further, and hopefully improves. In this regard, it seems clear that to make a certain progress here will also depend much on one’s path through life. For instance, someone whose second nature it is to search for knowledge—i.e., through studies, travels, or profound personal experiences—is likely to be a more nuanced decision maker than someone who prefers a less diverse way of living (Mieg, 2006). This does not mean that the former walk of life is better or worse than the latter, nor that a particular path through life by definition leads to particular skills or behavior. It merely suggests that someone who is used to dealing with mentally challenging issues or enjoys an environment where discussing such issues is common, will approach these issues and similar ones fairly well-considered. In a way, this thought holds a hidden plea for considering an individual’s biography as a cornerstone of decision-making abilities as well. Obviously, this leads to questions such as how to weigh crucial parts of an individual’s biography and how to value and operationalize them as clues for a *sufficient* decision-making ability. To eventually find legally usable answers to these questions, however, is a challenge. Nonetheless, it is vital to face these questions, the more so as they have a certain wit. In short, a mission for normative (law/ethics), social, and psychological scholars to join in multidisciplinary research into these matters lies ahead.

An interesting notion in this regard is the ‘unity of the individual’. While we continuously develop physically as well as mentally, and as we age ceaselessly (to some unmercifully), we tend to remain the same biographical being. In addition to this, we have a sense for what could be called the ‘continuity of the person’, which allows us, for instance, to relate to our past, to experience our present tense and to have expectations for our future. Furthermore, this ‘continuity of the person’ appears relevant to our personal identity, which is believed not only to refer to legally relevant characteristics such as nationality, name or family relations, but to authenticating signs such as language, sexual orientation, tribe, color, religious believes and normative convictions as well (Doek, 2006; Tobin & Todres, 2019).^18^ These signs may count as the actual cyphers of one’s personal identity and that is why these should not be overlooked when examining whether a minor’s terminal choice reflects his or her true inner conviction. Apart from that, it seems clear that to verify whether a decision constitutes what the individual involved truthfully desires is not the same as to determine whether this individual made the one and only right decision (as far as there is such a thing). Frankly, the basic idea of respect for autonomy, apart from considerate argumentations that emphasize a more detailed approach, is to comply with an individual’s authentic preference, especially when it concerns ultimately private (medical) decisions, rather than making sure that this individual achieves the arguably best decision (Schneider, 1998).

It is further noteworthy that in order to render legally relevant judgement on someone’s decision-making abilities, a distinction between static and dynamic utilizations to this idea of ‘personal continuity’ may be considered. In certain areas of law, an individual remains the same human being regardless of personal growth, while in others, due to ageing or developments in mental or social behavior, this human being is susceptible to personal change in a way that this becomes legally relevant and merits legal consequences. A judicially motivated static effect of this personal continuity may, for instance, be illustrated by an existing—yet, challenged—legal view (Schabas, 1996) on the workings of capital (Schabas, 2002) or life without parole (Rideau, 1992) sentences, upheld in several States of het USA.^19^ This view implies that if such sentences are imposed on someone who is legitimately convicted for terrible crimes, these sentences in time are rarely converted into less severe punishments on the ground that the convicted person ‘exists no more’ since the detainee has evolved into another personality during the years of persistent imprisonment. Hence, from an ethical or psychological viewpoint it may appear convincing to argue that the person who committed the crime is no longer identical with the person who has emerged after ‘doing time’ so that it would seem fair that the latter should serve a less severe punishment. Yet, this is hardly valid nor practicable under particular approaches of punishment under criminal law, which endorse the impossibility of interpersonal transmission of such sentences. This example shows that although changes in personal continuity might be apparent, these may not necessarily be legally relevant due to the static workings of (particular approaches to) penal law. Besides, to convert such a sentence on the dynamic temporal ground of authentic evolvements in one’s personal continuity would mean to distinguish between legally responsible personalities who are in fact incarnated in the same (bodily) individual (Roesch & Cook, 2017).^20^ From a legal point of view, the question remains: would it be acceptable to suspend, or even tone down individual legal responsibility for appalling acts—which obviously remain irrevocable—because of time-provoked personal evolvements within that individual? And while for the purpose of clarification this example of an inflexible legal attitude towards dynamics in one’s personal continuity applies to (actions by) adults, can there be good reason to attach legal consequences to apparent dynamics in the personal continuity of a hopelessly and unbearably suffering minor patient, particularly when it comes to assessing his/her wish to die? In view of the preference mentioned in paragraph 5.1 to consider combining chronological as well as non-chronological standards for reliably establishing competent decision-making by such a minor, prudency requires to take account of verifiable dynamics in this minor’s personal continuity too. Yet, where in older minors such dynamics are probably quite detectable, in minor of younger age this is far from evident. This also points at particular difficulties in using and interpreting such dynamics, for such lay ahead already, since in the Netherlands the extension of the laws on physician-assisted dying with regard to hopelessly and unbearably suffering sick minors below the age of 12 years, is currently debated. Whereas the Dutch Pediatricians Association tends to support such an extension, the Dutch Public Prosecutor’s Office is not convinced of its legal admissibility. With regard to possible additional values in noting relevant dynamics in a minor’s personal continuity, it may be argued that adopting this statutory extension, so that minors below this age in exceptional circumstances can be received in their request to part life, hardly needs consideration without a full clarity on the usability and applicability of the dynamics mentioned. This would also include proper understanding of the extent to which relevant dynamics in the personal continuity of a young minor can be identified.

### Towards assessment criteria for relevant dynamics in a minor’s personal continuity

Intuitively, there are credible motives for awarding legal significance to dynamic evolvements within the personal continuity of a medically troubled minor. However, which evolvements reflect such connotation and how are these to be valued in order to be instructive in the assessment of a minor’s *sufficient* ability to choose death over an agonizing existence? And, apart from that, do such evolvements contain components that are of special relevance in view of their temporality-related nature?

As for which personal evolvements can have legal significance, I tend to believe there are three areas strongly linked to individuality where such evolvements may be detected. To point at these areas does not cancel out the importance of other attributes of personal growth, such as the realm of learning or an inquisitive attitude towards narratives, nor does it imply any specific hierarchy between them. For, to identify these three areas is likely to go with justified open questions as to how these might interconnect or to what extent one may dominate or direct the other(s). Yet, for the moment, such matters are left aside. As to why these three particular areas seem relevant, I can only refer to my experiences as an alternate juvenile judge. During those years, I mainly dealt with issues of family law, including cases concerning the termination of parental authority, whether it is in the best interest of a child to remain in a foster family, the legal recognition of a child by its material father figure, rather than its irresponsible biological father, and the settlement of adoption. In many cases, through personal conversations, the preferences of minors involved were sought. In doing so, the Court gained a trustworthy impression of these minors’ capacity for discernment. Quite often, and also apparent in verbatim reports of such conversations, the minors involved demonstrated evolvements in the three areas mentioned below which imply ripened faculties for sound (enough) judgement.

The first area, as already suggested in the previous paragraph, concerns the framework of one’s biography. It seems obvious that at least parts of one’s biography can point at significant evolvements in one’s personal continuity. Such parts may correspond with personal events that have a substantial impact on the individual and his/her attitude towards oneself as well as others. General examples of such occurrences are easily given (i.e., death of a parent or grandparent, loss of relationships, threats to personal safety, the effect of serious disease within one’s family), but it is evident that these hardly affect every individual in a similar way. Therefore, a standardized list of impact-guaranteed events will not be useful. Besides, what occurrences in the relatively short life as a minor can have such an impact that it gravely influences the individual’s personality? And what if such life-events did not occur or were lived through less affectedly than one would generally expect? Surely, such objections are not without merit, but, evidently, it is not the ‘objective’ magnitude of an event but rather its subjective imprint (or the lack of it) on the minor’s constitution that can reveal a sort of ripened mental and emotional state of being. Interestingly, legally significant evolvements within one’s biography seem subjected to temporality as well. Their temporally determined nature at least seems to show from the fact that personal time after impactful events appears to be spend to a cathartic effect. To ‘consume’ time in such fashion at least amounts to an indicator for decision-making abilities that meet the ‘sufficient’-keystones of *understanding, appreciation, deciding* and *reasoning.*

A second area of dynamic evolvements within one’s personal continuity does not refer to particular occasions, but rather to a sound attitude when dealing with extraordinary situations. Such an attitude seems to originate from proceeding prudently. Defined as the ability to govern and discipline oneself by the use of reason, to practice prudence (Comte-Sponville, 1995)^21^ in our context means that the evolvements have taken root along the line of rationality, thus revealing one’s considerate approach to issues, and enabling outsiders to reconstruct and comprehend the involved individual’s line of thought. Prudence is a preferred expertise, a kind of practical wisdom when there is uncertainty, a risk for failure or an unknown perspective. In this regard, prudence is an instrumental quality, as it constitutes a precondition without which one would hardly know how to achieve a desired objective. And apart from the fact that it is a beneficial tool to interpret facts and circumstances, a prudent attitude is related to time as well, and sometimes even capable of saving time, as it facilitates taking account of the future—at least the one we are able or willing to face. In that sense, a prudent individual anticipates matters and does not only look at what happens or has happened, but also at what could happen (Comte-Sponville, 1995). This shows that prudent performances indeed involve a temporal component, which also feeds our ability to foresee. And because a preferred use of reason presupposes a sensibility to time-related perspectives, it can be fairly argued that prudence reflects a decision-making ability essential to how the ‘sufficient’-keystones of *understanding*, *appreciation*, *deciding* and *reasoning* are employed.

Of course, one can allege that it is difficult to measure whether a decision originates from enough prudence. This would support the need for an assessment standard of prudency. However much certainty such a standard would provide, one must admit that the nature of a prudent attitude is hardly demonstrable by the direction it indicates, but rather by an individual’s awareness that a considerate approach still leaves room for error. This means that prudency holds no guarantee for the ultimately correct decision, but enough for a decision to account for.

A third area of dynamic evolvements within one’s personal continuity pertains to one’s sensitivity to the perceptions and emotions of others. In displaying empathy, an individual gives an indication of the ability to share someone else’s feelings or experiences, by imagining what it would be like to be in that individual’s situation.^22^ This ability is a social competence, which in essence reflects a sensitivity to the need of others to be genuinely understood and be taken seriously in their views, thoughts and worries. In our context, a manifestation of empathy would involve an openness of the involved minor towards the perspectives of his/her parents, relatives, and close friends, but also to the insights of physicians, nurses, psychologists, etcetera. Nevertheless, a demonstration of empathy will probably not solve the issue faced by the agonized minor patient. Apart from that, to consider an empathic ability as a solid phenomenon, which must be demonstrated, could, in fact, unnecessarily burden the minor with additional worries about how to meet various expectations of others. On reflection, it rather looks as if having empathy is part of the ability to make prudent decisions. As mentioned above, prudency aims to get a grip on things and in trying so, one may want to consult confidants, so as to appreciate one’s assessments and to the effect of sharing sentiments, in certainty of not being alone in one’s existential struggle. Yet, for a minor to show empathy in such difficult circumstances is far from self-evident. The question is therefore: what is the gain of an empathic conduct by the minor involved? Could it be that such empathy above all would make others feel confident about the minor’s ability to decide prudently? To me at least this seems plausible, apart from the impression that a minor’s empathic evolvement could also bring others to an increased understanding of the wish to die and, perhaps eventually, to come to terms with it.

As for the temporal qualities of an empathic attitude, it does not suffice to assume that social sensibility usually—sometimes more and sometimes less—develops over time, for instance during the process of maturation. Such assumptions fall short, especially since minors concerned may not reach the corresponding calendar age. However, if empathy can reveal itself as a characteristic of a matured individual, who through evolvements has integrated this ability, this also would support the idea that chronological age should not be its sole decisive indicator. In consequence, this ability, if present sufficiently, could provide a clue for approving a wish to die, also if expressed by a minor. Yet, would this also mean that a minor who lacks a heightened sense of what moves others or for what others believe to be important, should be denied a request for a humane death? If so, the risk of inequity in decision-making validation might lie in wait. Besides, it fairly seems a token of empathy towards a minor facing the greatest agony a human individual can experience, that he or she is hardy expected to appreciate the thoughts and emotions of others in a way fitting to his or her own sorrow and despair. This, once again, suggests that a minor’s empathic nature seems above all relevant for others, so they may have good faith in the minor’s decision-making abilities. The minor is more likely to resign himself or herself to believing that the decision to part from life is made in liaising with the perceptions and emotions of those who are important to him/her.

A joint problem of all three areas addressed is how their dynamic evolvements in a minor’s personal continuity must be valued. To translate these evolvements into a useable assessment standard would require to operationalize them. However, these evolvements are difficult to quantify, because their value appears hard to establish by means of a formula or a mathematical equation. Consequently, their value may better to be determined by qualitative standards and objectified by qualitative research. This implies that scientific attempts to operationalize biography, prudence, and empathy as areas where dynamic evolvements in the personal continuity of a minor individual dwell, are to be drafted in line with qualitative research principles. I believe that such attempts should be encouraged, not only because they could increase our understanding of the feasibility of these areas as sources of assessment criteria for the decisional abilities of minors, but also since qualitative judgements are typical for the field of law. In fact, these judgements reflect an essential aspect of the legal discipline. For, as a well-known proverb states: law is no math!

### Relevant dynamics in a minor’s personal continuity in end-of-life legislation?

Finally, in how far were the Dutch and the Belgian legislator sufficiently aware of the temporality-related areas mentioned and has there been legal reason for them to note these areas when adopting the EA and the BEA? Here, a few remarks in reply.

The clarifications on the EA and the BAE in paragraph 4 have shown that both domestic regulations do not contain any reference to the temporality-related areas discussed in paragraph 5.3. The provisions in het Dutch EA dealing with death on a minor’s request only state as of which chronological age a minor can express a lawful wish to die and which procedural steps regarding the process of decision-making to grant such a wish, are then to follow. The fact that minors older than 11 years under Dutch law can be considered competent to express a terminal wish originates from a regulation in the Dutch Civil Code, concerning the medical treatment agreement between a physician and his/her patient. This regulation states that competent minors aged between 12 and 16 years, as well as their legal representative(s), must give consent for their medical treatment. The regulation provides no further instructions on how this minor’s competency to consent is to be established. In practice, the physician in charge decides about this, including which standards he/she sees fit for that purpose. In fact, Dutch law only imposes the condition that competency must be determined in agreement with applicable professional standards and current guidelines. In result, there is great trust in the professional skills of the physician involved to draw an accountable conclusion. Whether a physician merely checks the minor’s age, additionally examines some of his/her mental capacities or mostly uses a specific roadmap is presently not that clear.^23^ The same goes for whether Dutch physicians take proper notice of temporality-related areas of a minor’s personal continuity when deciding on a minor patient’s decision-making ability. Besides, up to date, no particular research into the specific competency of minor patients who express a wish to die—a competency to be distinguished from the one expected of minors who decide whether or not to undergo medical treatment—has been conducted in the Netherlands.^24^

Considerate arguments to involve temporality-related areas of a minor’s personal continuity when deciding on minor patients’ end-of-life decision-making abilities have been offered in the previous paragraphs. Nevertheless, it will probably take authoritative research, which would have to reveal that current ways to determine such abilities contain serious flaws and that a particular use of proposed standards, such as chronological combined with non-chronological ones, offers more reliable findings, to cause a significant change in the Dutch practice of establishing decision-making abilities in these minor patients. Since, in addition, current practice of euthanasia involving minors in the Netherlands reveals no particular problems, which could be traced back to negligent assessments of minor patient’s death wishes, leading to unlawful deaths of such minors, there are in fact no substantial legal motives for such a change whatsoever.

A far more explicit picture may be drawn for the BEA (Van Assche et al., 2019). As shown above, the Belgian regime specifies that accountable decision-making about end-of-life requests by a minor will not do without consulting a child- or youth-psychiatrist or a psychologist. Furthermore, the BEA describes what these professionals are expected to do, which is: to study the minor’s medical file, to examine him/her, to establish his/her decision-making capacities and to attests to all this in writing. Moreover, the consulted professionals must have special qualifications and must operate independent of the physician in charge. Besides, the nature of the opinions of these consultants is not merely advisory, but rather considered to have a binding character. All this testifies to the fact that the BEA way more than the Dutch EA explicates the exceptional responsibility of these professionals in reaching highly accountable decisions, also when dealing with establishing a minor patient’s required ‘capacity for discernment’. It also contributes to an increased awareness among the medical professionals involved; an awareness, that above all will keep them sensitive to possible weaknesses in their ‘routines’ and keep them critical towards the instruments and standards they use to fulfil their duties under the BEA. Interesting, in this regard, is that in Belgian expert literature (Van Assche et al., 2019), complying with the norm of ‘capacity for discernment’ has, apart for age, also been discussed in relation to autonomy and maturity. Here, a sense for the weight of other than chronological categories usable to assess decision-making skills of minors seems in progress. This may indicate, and here lies a lesson to be identified, that the search for reliable and accountable standards for establishing decision-making abilities in minor patients is underway and that opportunities to introduce temporality-related elements in the professional debate regarding the establishment of such abilities in minor patients lie ahead.

## Final remarks

The aim of this paper was to present an exploration of the possible meaning of temporality-related considerations for a highly sensitive issue in health law: end-of-life decisions in pediatrics. As was shown, the Netherlands and Belgium have adopted statutory regulations, which conditionally permit minor patients to express a legally relevant wish to die in circumstances of hopeless and unbearable suffering, due to severe and often untreatable medical conditions. In both jurisdictions, it is imperative that the minor patient concerned is able to choose for his/her death in a sufficiently considerate fashion.

A purpose of this exploration was to problematize how this ability is approached as well as determined from a current legal point of view in comparison to temporality-related building blocks of this ability, which are, however, hardly considered useable under statutory laws, also were domestic laws cover euthanasia and physician-assisted suicide in minors. This has been the starting point for reflections on the extent to which a minor patient’s ability to wish for his/her life to end interrelates with temporality, and whether certain areas of this ability can yet be legal significant. In this regard, it was discussed whether identified areas of temporality-related evolvements in the personal continuity of a minor patient can be (made) useable for assessing this patient’s end-of-life request.

It is obvious that the reflections presented are but an impetus for further thoughts and analyses on these matters. Still, the exploration undertaken has revealed some justifications for an intensified search for legal resonance in the addressed regions of temporality. To do so is worthwhile, as keystones of the legal assessment of a minor patient’s decision-making ability indicate plausible connections to three dynamic areas in a minor’s personal continuity. For many scholars, this may not come as a surprise. Yet, in the legal discourse, systematic attention for the additional value of temporality in view of the interpretation of a minor patient’s significant personality evolvements is lacking. This is a regret, since there is a need for efforts to advance in this barely explored angle of legal science, especially if we aim to grasp how time-connected sufficient legal understanding of a sick minor’s wish to die inevitably is.

## Footnotes

1 However, this is not always the case. An English court rejected a medical team’s advice to withdraw artificial ventilation from a 18-month-old baby, MB, and agreed with the infant’s parents’ wish to continue ventilation, which they considered to be in the best interest of their child. See An NHS Trust v. MB & Anor (2006) EWHC 507 (Fam). In the American case of Baby K a physician’s order to withhold life-saving mechanical ventilation from a newborn anencephalic infant was regarded inadmissible, as it violated the federal statutory prohibition of disability discrimination. See In re Baby K, 832 F. Supp. 1022, ED Va 1993; In re Baby K, 832 F. Supp. 1022, ED Va 1993 as well as US Supreme Court, 513 US 825 resp. 115 S. Ct. 91; 63 USLW 3258, October 3, 1994.

2 https://www.who.int/health-topics/palliative-care (Final consultation on September 9th 2022).

3 VAD will be possible in Tasmania as of 23 October 2022, in South Australia as of 31 January 2023, in Queensland as of 1 January 2023 and in New South Wales as of 28 November 2023. In the Northern Territory and the Australian Capital VAD is expected to remain prohibited. See https://end-of-life.qut.edu.au/assisteddying (Final consultation on September 9th 2022).

4 Bundesverfassungsgericht, Urteil des Zweiten Senats vom 26. Februar 2020—2 BvR 2347/15. See https://www.bundesverfassungsgericht.de/e/rs20200226_2bvr234715.html

5 Act of 12 April 2001, *Governmental Gazette* 2001, 194.

6 See RERC-Judgement 2022–72 at https://www.euthanasiecommissie.nl/uitspraken-en-uitleg/p-2022/documenten/publicaties/oordelen/2022/2022-061-tm-2022-80/oordeel-2022-072

7 At https://english.euthanasiecommissie.nl/

8 Act of 28 May 2002, *Belgian Governmental Gazette* 22 June 2002, valid as of 23 September 2002. The 2002 version of the Act is reproduced in *European Journal of Health Law* 2003/3, 329–335.

9 Act of 28 February 2014 (W2014-02–28/03, art. 2, 003) valid as of 22 March 2014.

10 Belgian Constitutional Court, Judgement no. 153/2015, 29 October 2015. See https://www.const-court.be/public/e/2015/2015-153e.pdf.

11 The ECtHR concluded that Belgium violated these obligations by omitting to safeguard the independence of the FCECE when reviewing this case of euthanasia performed on a woman suffering from depression, and by spending too much time (= in total four years and eight months) on criminal investigations following complains about this case. See Mortier v. Belgium, Judgement of 4 October 2022, (case nr. 78017/17) at.

12 See https://overlegorganen.gezondheid.belgie.be/sites/default/files/documents/fcee-reactie-cehrm-221014_0.pdf

13 All reports available at http://overlegorganen.gezondheid.belgie.be/nl/advies-en-overlegorgaan/commissies/federale-controle-en-evaluatiecommissie-euthanasie

14 See https://overlegorganen.gezondheid.belgie.be/sites/default/files/documents/fcee-cijfers-2020_persbericht.pdf. Press release of 2 March 2021. This document, however, holds an obvious typographical error, as it repeats the reference to the case concerning a minor in 2019, but omits to report about the actual situation on minors in 2020. (Final consultation on 22 November 2022. At that moment, the expected Tenth Annual Report of the FCECE, showing all data covering 2020–2021 was not yet published).

15 See https://overlegorganen.gezondheid.belgie.be/sites/default/files/documents/fcee-cijfers-2021_persbericht-totaal.pdf*.* Press release of 31 March 2022.

16 At http://overlegorganen.gezondheid.belgie.be/nl/advies-en-overlegorgaan/commissies/federale-controle-en-evaluatiecommissie-euthanasie (Final consultation on 22 November 2022).

17 See note 19.

18 Article 8 of the UN-Convention on the Rights of the Child (CRC) mentions that States Parties must respect the child’s right to preserve his or her identity. If a child is illegally deprived of some or all of the elements of his or her identity, States Parties must provide appropriate assistance and protection, with a view to re-establishing speedily his or her identity.

19 See https://deathpenaltyinfo.org/policy-issues/sentencing-alternatives/life-without-parole

20 Still, this is less absolute than it may seem, Forensic mental health care shows examples of psychiatric pathology (= i.e. personality disorders, such as paranoid-, schizoid-, schizotyped-, borderline, narcissistic personality disorders) in persons who committed crimes, but whose ‘dominant personality’ is sometimes hard to relate to the particular crime.

21 Prudence (in Latin ‘prudentia’; in Greek ‘phronèsis;) means ‘seeing ahead’ and is regarded—besides temperance, fortitude and justice—as a cardinal virtue.

22 As in https://dictionary.cambridge.org/dictionary/english/empathy

23 Remarkably though, earlier research showed, among other things, that Dutch physicians tend to consider a minor patient to be incompetent for decision-making in medical care matters in situations where the minor fairly disagrees with a physician’s treatment proposal. See Dute et al. (2000).

24 A study, supported by the Dutch Ministery of Health Care and conducted by the Dutch Pediatric Association, which—among other things—intended to find clues in this regard did not succeed, since no minors or parents concerned and willing to be interviewed on the matter were found. See Brouwer et al. (2019).
